# Factors associated with daily life physical activity in patients with asthma

**DOI:** 10.1002/hsr2.84

**Published:** 2018-08-15

**Authors:** Florence Hennegrave, Olivier Le Rouzic, Stéphanie Fry, Hélène Behal, Cécile Chenivesse, Benoit Wallaert

**Affiliations:** ^1^ Service de Pneumologie et ImmunoAllergologie, Centre de Référence constitutif des Maladies Pulmonaires Rares, Univ. Lille CHU Lille, Hopital Calmette Lille France; ^2^ Department of Biostatistics, Univ. Lille, EA 2694‐Santé Publique: Epidémiologie et Qualité des Soins CHU Lille Lille France

**Keywords:** anxiety, asthma, asthma control, daily life physical activity, depression, severity

## Abstract

**Background and Objectives:**

Little is known about the consequences of asthma on daily life physical activity (DL_PA_). The aim of this study was to evaluate DL_PA_ and determine its relationship to clinical and functional parameters in patients with asthma.

**Methods:**

This was a single‐center prospective study of DL_PA_ conducted between May 2015 and June 2016 in northern France. Fifty‐one adult patients with asthma and 36 healthy control subjects were enrolled. Four DL_PA_ parameters were assessed for 5 consecutive days with a physical activity monitor: number of steps walked per day (SPD), total energy expenditure (EE, in kcal/day), EE spent in physical activity requiring ≥3 metabolic equivalents (METs), and time (min) spent in activities requiring ≥3 METs. Clinical characteristics, pulmonary function tests, 6‐minute walk test, and four questionnaires (modified Medical Research Council [mMRC] for dyspnea, asthma control test [ACT], quality of life [AQLQ], and hospital anxiety and depression scale [HADS]), were evaluated. Comparisons of DL_PA_ parameters between the two groups were performed using an analysis of covariance adjusted for age, sex, and body mass index (BMI). Relationships between DL_PA_ parameters and patient characteristics were assessed in multivariable linear regression models.

**Results:**

Compared with patients with mild/moderate asthma, those with severe asthma had lower mean (± standard deviation) forced expiratory volume in 1 s (FEV_1_) (66 ± 24 vs 94 ± 15% predicted, *P* < 0.001), ACT score (16.7 ± 4.5 vs 19.8 ± 4.2, *P* = 0.015), and AQLQ score (157 ± 40 vs 184 ± 33, *P* = 0.012). There were no significant differences between the two groups in SPD (6560 ± 3915 vs 8546 ± 3431; adjusted *P* = 0.95), EE in physical activity requiring ≥3 METs (620 ± 360 vs 660 ± 140 kcal/day; *P* = 0.86), time spent in activities requiring EE ≥3 (120 ± 54 vs 121 ± 32 min/day; *P* = 0.69), or total EE (2606 ± 570 vs 2666 ± 551 kcal/day; *P* = 0.80). These four DL_PA_ measures showed strong inter‐parameter correlations in patients with asthma (*r* = 0.37–0.95, all *P* < 0.01). All four parameters were lower in the patients with asthma group than in the control group: SPD, 7651 ± 3755 vs 11704 ± 4054 (adjusted *P* < 0.001); EE in activities requiring ≥3 METs, 642 ± 360 vs 852 ± 374 kcal/day (adjusted *P* = 0.041); time spent in activities requiring ≥3 EE, 120 ± 73 vs 189 ± 85 min (adjusted *P* = 0.005); and total EE, 2639 ± 555 vs 2746 ± 449 kcal/day (adjusted *P* = 0.007). In the patients with asthma group, the number of SPD correlated with age, FEV_1_, mMRC score, 6‐minute walk test distance, and HADS scores, but not with BMI or ACT test score. Using multivariate analysis, the number of SPD was associated with only age, anxiety, and FEV_1_, whereas total EE was associated with mMRC score and BMI.

**Conclusion:**

Age, anxiety, and FEV_1_ were significantly associated with the number of SPD in patients with asthma. Addressing anxiety should be further studied as way to attempt to increase physical activity in patients with asthma.

## 1 INTRODUCTION

Asthma is a chronic inflammatory disease of the airways that is estimated by the World Health Organization to affect ~300 million people worldwide.^1^ According to the 2015 Global Initiative for Asthma, the degree to which physical activity is limited should be considered when assessing asthma control.^1^ Physical activity is an important clinical parameter related to morbidity and mortality in many chronic diseases,^2^, ^3^ and higher levels of physical activity are associated with better lung function in asthma patients.^4^ However, a recent review suggests that individuals with asthma are less likely than healthy subjects to engage in physical activity, despite its beneficial effects on symptom management, lung function, and mental health; conversely, physical inactivity was found to be associated with negative health consequences.^5^ Patients with chronic obstructive pulmonary disease (COPD) also have low levels of physical activity, but this is not reflected in the clinical characteristics commonly used to determine disease severity.^6^, ^7^

Few studies have objectively evaluated daily life physical activity (DL_PA_) levels in patients with asthma. Recently, Bahmer et al^8^ and Cordova‐Rivera et al,^9^ using objective DL_PA_ parameters, also reported that physical activity was reduced in severe asthma patients. van't Hul et al studied a large group of adults with bronchial asthma and found lower than average values for the number of steps per day, total energy expenditure (EE), and time spent in intensive physical activity; however, the study did not include patients with severe asthma.^10^ In contrast, Sousa et al found no correlation between DL_PA_ and asthma severity in children; indeed, DL_PA_ levels were similar for children with good asthma control and those without asthma.^11^ With these studies in mind, we sought to identify some of the factors, including anxiety and depression, that might influence DL_PA_ in adult patients with severe or mild/moderate asthma. We examined the relationships between DL_PA_ and the results of pulmonary function tests, the 6‐minute walk test (6MWT), and of four questionnaires: the asthma control test (ACT), asthma quality of life questionnaire (AQLQ), modified Medical Research Council (mMRC) dyspnea questionnaire, and the hospital anxiety and depression scale (HADS) questionnaire.

## 2 METHODS

### 2.1 Patients

We enrolled 51 consecutive patients with asthma (28 mild/moderate, 23 severe) who were referred to our hospital between December 2013 and January 2015 for routine follow‐up. Severe asthma was defined as “asthma which requires treatment with high dose inhaled corticosteroids plus a second controller (and/or systemic corticosteroids) to prevent it from becoming ‘uncontrolled’ or which remains ‘uncontrolled’ despite this therapy.”^12^ The control group included 36 healthy subjects who were students or relatives of employees at the hospital. All control subjects had normal spirometry results. None of the patients or control subjects was engaged in exercise training programs prior to the study. All individuals gave informed consent. Approval for the use of these data was provided by the Institutional Review Board of the French Learned Society for Pulmonology (CEPRO 2011‐039).

### 2.2 Pulmonary function tests

Forced vital capacity (FVC), forced expiratory volume in 1 s (FEV_1_), and total lung capacity were measured by spirometry and plethysmography with a Jaeger‐Masterlab® cabin. Values are expressed as the percentage of the predicted normal value.^13^, ^14^, ^15^ The 6MWT was performed in accordance with international recommendations.^16^

### 2.3 Assessment of DL_PA_

Subjects were equipped with a physical activity monitor (SenseWear® Pro armband and SenseWear software version 8.0; BodyMedia Inc., Pittsburgh, PA, USA) and instructed to wear the device continuously, except while showering or bathing, for 5 consecutive days (3 weekdays and 2 weekend days). The device was positioned on the upper right arm, at the midpoint between the acromion and the olecranon. This multiaxial device has been validated in diverse populations, including patients with chronic diseases.^8^, ^17^, ^18^, ^19^ DL_PA_ was assessed by measuring four parameters: the average number of steps per day (SPD), the average time (min/day) spent in activities with an estimated EE of ≥3 metabolic equivalents (METs), the average EE spent in activities requiring ≥3 METs (kcal/day), and the average total daily EE (kcal/day). METs reflect the energy cost of physical activity as a multiple of the patient's resting metabolic rate.^20^ EE ≥ 3.0 METs is considered to be at least moderate activity.^21^

### 2.4 Evaluation of dyspnea

Subjects reported their perception of dyspnea occurring during DL_PA_ using the mMRC self‐administered questionnaire, which was assessed at the time of evaluation. The mMRC questionnaire is a unidimensional assessment of how dyspnea/breathlessness limits activity and affects functional ability, employment (disability), quality of life, and health status.^22^ The questionnaire was developed in the 1950s by the Medical Research Council with the aim of detecting pneumoconiosis in coal workers, and was later standardized by Fletcher.^23^ Since then, it has been validated in several chronic respiratory diseases, including asthma.^24^ The five questions assess perceived breathlessness on a scale from 0 (not troubled by breathlessness except during strenuous exercise) to 4 (very severe dyspnea: too breathless to leave the house or breathless when dressing or undressing).

### 2.5 Asthma control test (ACT)

The ACT survey is a self‐administered questionnaire consisting of five items assessing asthma symptoms (daytime and nocturnal), use of rescue medications, and the effect of asthma on daily functioning.^25^ Each item includes five response options corresponding to a five‐point Likert‐type rating scale. Responses for each of the five items are summed to yield a score ranging from 5 (poor control) to 25 (complete control). An ACT score > 19 indicates well‐controlled asthma.

### 2.6 Quality of life

The AQLQ comprises 32 items and is designed to identify the areas of functioning impaired by asthma in adult patients.^26^ It measures four domains: activity limitation (11 items), emotional function (five items), exposure to environmental stimuli (four items), and symptoms (12 items). The patient describes his/her experience with each domain in the previous 2 weeks, using a seven‐point scale (1, severely impaired to 7, not impaired at all). Higher scores indicate better quality of life.

### 2.7 Evaluation of anxiety and depression

The HADS questionnaire was designed to identify and quantify the two most common forms of psychological disorders in medical patients, anxiety and depression.^27^ Both subscales range from 0 to 21, with a score of ≥8 indicating clinically relevant symptoms.

### 2.8 Statistical analysis

Statistical analysis was performed using SAS statistical software, version 9.3 (SAS Institute Inc., Cary, NC) and GraphPad Prism 5 (GraphPad Software Inc., La Jolla, CA). Normal distribution of the data was checked graphically and using the Shapiro‐Wilk test. Continuous variables are expressed as mean ± standard deviation (SD) for normally distributed data or median and range [min‐max] for non‐normally distributed data. Categorical variables are presented as frequencies and percentages. Categorical parameters were compared between healthy subjects and asthma patients by the Chi‐square test or Fisher's exact test. Continuous variables were compared between the two groups by Student's *t*‐test or the Mann‐Whitney *U*‐test. The same tests were used to compare patients with mild/moderate and severe asthma. Comparisons of DL_PA_ parameters between two groups were performed after adjustment on pre‐specified confounding factors (age, sex, and body mass index [BMI]) using analysis of covariance. Correlations between the four DL_PA_ parameters in asthma patients were assessed by calculating Pearson's correlation coefficients. Factors associated with each DL_PA_ parameter in patients with asthma were assessed using univariate and multivariate analyses. Candidate factors were age, BMI, FEV_1_, FVC, FEV_1_/FVC, ACT score, AQLQ score, HADS scores, mMRC score, and the 6MWT distance. Univariate analysis was performed by calculating Pearson's correlation coefficients. Univariate predictors (at *P* < 0.1) were entered into multivariable linear regression models (one per DL_PA_ parameter). Prior to the multivariable analysis, we first checked for the absence of co‐linearity between candidate variables by calculating the variation inflation factor^28^ and then tested all possible first‐order interactions between candidate factors in separate linear regression models. To simplify the multivariable models, a backward‐stepwise selection procedure was used with a removal criteria of *P* = 0.05. Statistical testing was done at the two‐tailed α level of 0.05.

## 3 RESULTS

The characteristics of the patients and healthy control subjects are summarized in Table 1. The two groups were comparable in terms of sex, age, and smoking status, whereas patients had significantly lower FEV_1_, FVC, and FEV_1_/FVC ratio than the healthy controls (mean ± SD: 82 ± 24 vs 104 ± 4% predicted, *P* < 0.001; 99 ± 18 vs 106 ± 3% predicted, *P* = 0.010; and 68 ± 15 vs 79 ± 2%, respectively, *P* < 0.001). Patients with severe asthma were older than those with mild/moderate asthma (mean ± SD: 54 ± 16 vs 34 ± 11 years, *P* < 0.001) and had significantly lower FEV_1_ (mean ± SD: 66 ± 24 vs 94 ± 15% predicted, *P* < 0.001), FVC (mean ± SD: 89 ± 17 vs 106 ± 15% predicted, *P* < 0.001), and FEV_1_/FVC (mean ± SD: 60 ± 16 vs 75 ± 9%, *P* < 0.001), but there were no significant differences in sex ratio, BMI, history of smoking, or HADS scores. Patients with severe asthma also had lower ACT scores (mean ± SD: 16.7 ± 4.5 vs 19.8 ± 4.2, *P* = 0.015), AQLQ scores (mean ± SD: 157 ± 40 vs 184 ± 33, *P* = 0.012), shorter 6MWT distance (mean ± SD: 462 ± 94 vs 608 ± 88 m, *P* < 0.001), and higher mMRC dyspnea scores reflecting greater dyspnea upon effort (median [min‐max]: 1 [0‐4] vs 0 [0‐1], *P* < 0.001) compared with patients with mild/moderate asthma.

**Table 1:** Characteristics of healthy control subjects and asthma patients

Characteristic	Healthy Controls (*n* = 36)	All Asthma Patients (*n* = 51)	*P* Value	Mild/Moderate Asthma (*n* = 28)	Severe Asthma (*n* = 23)	*P* Value
Male, *n* (%)	12 (33)	21 (41)	0.46^a^	13 (46)	8 (35)	0.40^a^
Age, years	45 ± 16	43 ± 17	0.58^b^	34 ± 11	54 ± 16	<0.001^b^
BMI, kg/m^2^	23 ± 3	26 ± 5	0.002^b^	25 ± 3	27 ± 6	0.10^b^
FEV_1_, % predicted	104 ± 4	82 ± 24	<0.001^b^	94 ± 15	66 ± 24	<0.001^b^
FVC, % predicted	106 ± 3	99 ± 18	0.010^b^	106 ± 15	89 ± 17	<0.001^b^
FEV_1_/FVC, %	79 ± 2	68 ± 15	<0.001^b^	75 ± 9	60 ± 16	<0.001^b^
Atopy, *n* (%)	NA	44 (86)	‐	27 (96)	17 (74)	0.037^c^
Age at diagnosis, years	NA	23 ± 14	‐	21 ± 11	24 ± 16	0.50^b^
Current smokers, *n* (%)	7 (19)	11 (21)	0.56^a^	7 (25)	4 (17)	0.73^c^
Smoking, pack‐year	4 [1‐35]	6 [1‐67]	‐	4 [1‐11]	20 [3‐50]	‐
Dyspnea, mMRC score	NA	1 [0‐4]	‐	0 [0‐1]	1 [0‐4]	<0.001^d^
ACT score	NA	18.4 ± 4.5	‐	19.8 ± 4.2	16.7 ± 4.5	0.015^b^
6MWT distance, m	NA	543 ± 116	‐	608 ± 88	462 ± 94	<0.001^b^
HADS score, depression	NA	3.8 ± 3.0	‐	3.3 ± 2.5	4.4 ± 3.5	0.18^b^
HADS score, anxiety	NA	7.4 ± 3.5	‐	7.2 ± 3.5	7.7 ± 3.5	0.62^b^
AQLQ score	NA	172 ± 38	‐	184 ± 33	157 ± 40	0.012^b^

a Chi‐square test.

b Student's *t*‐test.

c Fisher's exact test.

d Mann‐Whitney U‐test.

Data are presented as the mean ± SD except for smoking and mMRC score, which are median [min‐max].

Abbreviations: 6MWT, 6‐minute walk test; ACT, asthma control test; AQLQ, asthma quality of life questionnaire; BMI, body mass index; FEV_1_, forced expiratory volume in 1 s; FVC, forced vital capacity; HADS, hospital anxiety and depression scale; mMRC, modified Medical Research Council questionnaire; NA, not applicable.

**Figure 1:**
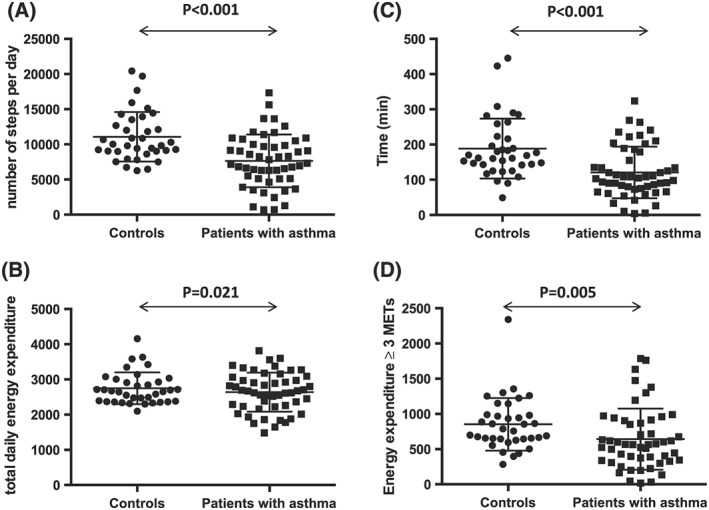
Comparison of daily life physical activities in asthma patients and healthy control subjects. A, Number of steps per day; B, total daily energy expenditure; C, duration of physical activity ≥3 METs per day; and D, energy expended during physical activity ≥3 METs in healthy controls (*n* = 36) and asthma patients (*n* = 51). Errors bars indicate mean ± SD. Differences were analyzed using Student's *t*‐test

As shown in Table 2 and Figure 1, patients with asthma walked significantly fewer SPD than the control subjects (mean ± SD: 7651 ± 3755 vs 11704 ± 4054, *P* < 0.001) and spent significantly less time and effort in physical activities, as demonstrated by their lower total daily EE (mean ± SD: 2639 ± 555 vs 2746 ± 449 kcal/day, adjusted *P* = 0.007), lower EE ≥ 3 METs (mean ± SD: 642 ± 360 vs 852 ± 374 kcal/day, adjusted *P* = 0.041), and shorter time spent in at least moderate activity (mean ± SD: 120 ± 73 vs 189 ± 85 min/day, adjusted *P* = 0.005).

**Table 2:** Daily physical activity in healthy control subjects and patients with asthma

Parameter	Health Controls	Asthma Patients	*P* Value	Adjusted *P* Value*
	(*n* = 36)	(*n* = 51)		
Number of steps per day	11 704 ± 4054	7651 ± 3755	<0.001	<0.001
Total energy expenditure, kcal/day	2746 ± 449	2639 ± 555	0.021	0.007
Energy expenditure ≥3 METs, kcal/day	852 ± 374	642 ± 360	0.005	0.041
Duration of physical activity ≥3 METs, min/day	189 ± 85	120 ± 73	<0.001	0.005

Data are presented as the mean ± SD.

* Adjusted for age, sex, and body mass index.

Abbreviation: METs, metabolic equivalents.

As shown in Table 3, none of the DL_PA_ parameters were significantly different between the severe and mild/moderate asthma patient groups. However, for the whole patient cohort (*n* = 51), there were strong correlations between all of the DL_PA_ parameters (Pearson's *r* 0.56‐0.95, *P* < 0.001 for all correlations), with the exception of a weak correlation between the number of SPD and total daily EE (*r* = 0.37, *P* = 0.008; Table 4).

**Table 3:** Daily physical activity in patients with asthma

Parameter	Mild/Moderate Asthma	Severe Asthma	*P* Value	Adjusted *P* Value*
	*n* = 28	*n* = 23		
Number of steps per day	8546 ± 3431	6560 ± 3915	0.06	0.95
Total energy expenditure, kcal/day	2666 ± 551	2606 ± 570	0.70	0.80
Energy expenditure ≥3 METs, kcal/day,	660 ± 140	620 ± 360	0.75	0.86
Duration of physical activity ≥3 METs, min/day	121 ± 32	120 ± 54	0.95	0.69

Data are presented as the mean ± SD.

* Adjusted for age, sex, and body mass index.

Abbreviation: METs, metabolic equivalents.

**Table 4:** Correlation between the four parameters of daily life physical activity in patients with asthma (*n* = 51)

Parameter	Total energy Expenditure (kcal/day)	Energy Expenditure ≥3 METs (kcal/day)	Duration of Physical Activity ≥3 METs (min/day)
Number of steps per day	*r* = 0.37^a^ *P* = 0.008	*r* = 0.56 *P* < 0.001	*r* = 0.63 *P* < 0.001
Total energy expenditure	‐	*r* = 0.72 *P* < 0.001	*r* = 0.56 *P* < 0.001
Energy expenditure ≥3 METs	‐	‐	*r* = 0.95 *P* < 0.001

a Pearson's correlation coefficients.

Abbreviation: METs, metabolic equivalents.

In the asthma patient group, the mean number of SPD correlated with age (*r* = −0.45, *P* < 0.001), FEV_1_ (*r* = 0.41, *P* = 0.003), FEV_1_/FVC (*r* = 0.50, *P* < 0.001), mMRC score (*r* = −0.39, *P* = 0.004), 6MWT distance (*r* = 0.38, *P* = 0.007), HADS anxiety score (*r* = −0.30, *P* = 0.035). There was no significant correlation between the mean number of SPD and HADS depression score (*r* = −0.26, *P* = 0.070) (Table S1). Interactions between age and FEV_1_, age and anxiety, and FEV_1_ and anxiety on the number of SPD were significant at *P* < 0.1 and were included in the multivariable model together with the individually significant factors. After backward‐stepwise selection, only age, interactions between age and FEV_1_, and interactions between FEV_1_ and anxiety were associated with the number of SPD (*R* ^2^ = 0.51; Table 5).

**Table 5:** Multivariable linear regression analysis of factors affecting the number of steps per day in patients with asthma

Factor	Estimate	SE	*P* Value	Partial *R* ^2^ (%)
Age	−309.0	85.3	<0.001	24
FEV_1_	8.6	61.3	0.89	3
HADS score, anxiety	793.4	431.2	0.07	9
Age × FEV1^a^	3.0	1.1	0.011	6
FEV1 × HADS score, anxiety	−14.0	5.2	0.010	8

a “×” indicates interaction.

Abbreviations: FEV_1_, forced expiratory volume in 1 s; HADS, hospital anxiety and depression scale; SE, standard error.

While total EE correlated with the mMRC score (*r* = −0.30, *P* = 0.03), we did not observe significant correlations between total EE and HADS anxiety score (*r* = −0.27, *P* = 0.058) or BMI (*r* = 0.26, *P* = 0.063). No significant interactions between these parameters and total EE were found. In multivariate analysis, only BMI and mMRC score remained significantly associated with the total EE (Table S2).

The EE from activity requiring ≥3METs showed no significant correlation with HADS anxiety score (*r* = −0.27, *P* = 0.057) or mMRC score (*r* = −0.25, *P* = 0.077), and there was no interaction between these two parameters. In multivariate analysis, neither parameter remained significantly associated with EE ≥ 3 METS (Table S3).

No significant correlation was observed between the time spent in moderate activity (≥3 METs) and BMI (*r* = −0.25, *P* = 0.081), mMRC score (*r* = −0.24, *P* = 0.088), or HADS anxiety score (*r* = −0.24, *P* = 0.094). No interactions were found in bivariate analysis. In multivariate analysis, only mMRC score was significantly associated with the time spent in moderate activity (Table S4).

## 4 DISCUSSION

The results of this study show that DL_PA_ is decreased in adult patients with asthma, and that the number of SPD is associated with age, anxiety, and FEV_1_. Currently, there are two methods of evaluating physical activity levels: subjective methods such as questionnaires and diaries, and objective methods such as motion sensors, as used here to assess DL_PA_. Although no studies have yet directly compared objective and subjective measurements of physical activity, research in other fields suggests that the perception of symptoms does not rigorously correlate with sensory stimuli. For instance, the perception of dyspnea intensity by asthma patients is not strictly proportional to bronchial obstruction; similarly, the perception of asthma control by the patient and medical staff differ markedly. With regard to the duration of activity monitoring necessary for accurate assessment, a review of studies measuring physical activity in adults with an accelerometer determined that 3 to 5 days of monitoring were required to obtain reliable measurements.^29^ In a recent study of COPD patients, physical activity was assessed over 10 days, and the data were evaluated from the last seven daily records.^30^ The data presented here for asthma patients were collected over 5 consecutive days and were similar for weekdays and weekend days. Thus, the duration of data collection in our study is in line with the recommendation. Interestingly, the number of SPD walked by patients with severe asthma in our study was similar to that reported by Bahmer et al^8^ and Cordova‐Rivera et al for patients with severe asthma.^9^

Most previous studies examining associations between physical activity limitations and asthma control used subjective measures of DL_PA_. Recently, van't Hul et al reported that a low level of habitual physical activity was associated with poor asthma control^10^; however, the correlations between asthma control scores and physical activity were weak. Similarly, older adults with poor asthma control were nearly twice as likely as those with well‐controlled asthma to have limitations in DL_PA._ 31 Our results are in agreement with a study with adolescent asthma patients demonstrating that physical activity levels were not dependent on asthma severity.^32^ Moreover, DL_PA_ was not influenced by asthma control per se in our patient group. These results are in agreement with Yiallouros et al, who reported that schoolchildren (aged 8‐9 years) with active or inactive asthma had similar levels of objectively assessed physical activity.^33^

We found that impairment of FEV_1_, which is representative of the magnitude of bronchial obstruction in asthma, was associated with the number of SPD. Bahmer reported that FEV_1_ and peak expiratory flow were poor markers of physical activity in asthma patients, and they also found that reduced DL_PA_ was associated with impulse oscillometric airway resistance and small airway dysfunction,^8^ which was not evaluated in our study. Interestingly, no consistent association has been found between active lifestyle/physical inactivity and pulmonary function (after adjustment for confounding factors) in healthy subjects, lung‐healthy adolescents,^34^ or adult smokers.^35^ In our study, the number of SPD was not associated with BMI, as previously reported for non‐obese individuals.^36^ However, we did detect a correlation between BMI and total EE, and this was stronger after adjustment for mMRC score. Total EE includes the EE not only during physical activity but also at rest. Resting EE is the sum of the energy expended by a fasted individual at rest in a thermo‐neutral environment plus the thermic effect of feeding. Thus, body composition, ethnicity, physical fitness level, hormonal status, and multiple environmental factors can all influence total EE,^37^ which might explain the correlation between total EE and BMI detected here.

The number of SPD is also influenced by non‐physiological factors, including depression and anxiety.^38^, ^39^, ^40^, ^41^ We found a significant correlation between SPD and depression and anxiety scores in our asthma patients, although, interestingly, only age, anxiety, and FEV_1_ showed significant associations with the number of SPD. Similarly, Vermeulen et al recently reported that psychological burden was the major activity‐limiting factor for asthma patients.^42^ According to Herrmann,^43^ it is unlikely that age affects HADS scores. Indeed, a recent study of 176 Chinese asthma patients found that age did not have a major influence on anxiety.^44^ DL_PA_ is significantly lower in depressed compared with non‐depressed COPD patients,^45^ and anxiety is highly prevalent among asthma patients,^46^, ^47^, ^48^ where it is associated with more exacerbations,^49^ more hospital readmissions,^50^ and poorer asthma control.^51^, ^52^ The molecular mechanisms responsible for anxiety are not well understood and might involve abnormal neural processing of stimuli.^53^, ^54^ Therefore, it is possible that asthma patients avoid exercise for fear of triggering symptoms,^55^ which leads to a sustained overall aversion to exercise.^56^ Studies of patients with other chronic diseases have failed to demonstrate that increases in DL_PA_ can reduce symptoms of anxiety and depression.^57^, ^58^, ^59^ However, it remains to be determined whether treatment for anxiety (medication or supportive psychotherapy) can increase DL_PA_ in asthma patients. Encouragingly, two studies reported that asthma patients participating in a pulmonary rehabilitation program were able to increase their physical activity without exacerbating asthma, and in fact, it significantly improved their quality of life.^60^, ^61^ However, anxiety and depression were not evaluated in those studies.

The main limitation of our study is the small sample size. Therefore, we may have overlooked several interactions/associations due to inadequate statistical power. In addition, we acknowledge that we cannot exclude a degree of overfitting in the multivariate analyses given the number of variables analyzed. Further larger studies are warranted to confirm our findings.

## 5 CONCLUSIONS

Age, anxiety, and FEV_1_ are associated with the number of SPD in patients with asthma, who show reduced DL_PA_ compared with healthy subjects. Addressing anxiety should be further studied as way to attempt to increase physical activity in patients with asthma.

## CONFLICT OF INTEREST

For each author, no significant conflicts of interest exist with any companies or organizations whose products or services are mentioned in this article.

## AUTHOR CONTRIBUTIONS

Conceptualization: Florence Hennegrave, Stéphanie Fry, Benoit Wallaert

Formal Analysis: Hélène Behal, Florence Hennegrave, Olivier Le Rouzic

Investigation: Florence Hennegrave, Stéphanie Fry, Benoit Wallaert

Writing – original draft: Florence Hennegrave

Writing – review and editing: Florence Hennegrave, Stéphanie Fry, Cécile Chenivesse, Benoit Wallaert

## Supporting information

Table S1. Correlation coefficients between the four parameters of daily life physical activity and characteristics of patients with asthma (*n* = 51)
Table S2. Multivariable linear regression analysis of factors affecting the total energy expenditure (kcal/day) in patients with asthma
Table S3. Multivariable linear regression analysis of factors affecting energy expenditure ≥3METs (kcal/day) in patients with asthma
Table S4. Multivariable linear regression analysis of factors affecting the duration of physical activity ≥3METs (min/day) in patients with asthma
