# Supplementary material for: Factors associated with daily life physical activity in patients with asthma
Source: Health Sci Rep. 2018 Aug 15;1(10):e84. doi: 10.1002/hsr2.84 (PMC6266451; doi:10.1002/hsr2.84)
Supplement: Supplementary file 1 — Table S1. Correlation coefficients between the four parameters of daily life physical activity and characteristics of patients with asthma (n = 51) Table S2. Multivariable linear regression analysis of factors affecting the total energy expenditure (kcal/day) in patients with asthma Table S3. Multivariable linear regression analysis of factors affecting energy expenditure ≥3METs (kcal/day) in patients with asthma Table S4. Multivariable linear regression analysis of factors affecting the duration of physical activity ≥3METs (min/day) in patients with asthma [file HSR2-1-e84-s001.docx]

Supplementary Tables

Table S1. Correlation coefficients between the four parameters of daily life physical activity and characteristics of patients with asthma (*n* = 51)

|  | Number of steps per day | | Total energy expenditure (kcal/day) | | Energy expenditure ≥3METs (kcal/day) | | Duration of physical activity ≥3METs (min/day) | |
| --- | --- | --- | --- | --- | --- | --- | --- | --- |
| Parameter | *r* * | *P* value | *r* | *P* value | *r* | *P* value | *r* | *P* value |
| Age | −0.45 | <0.001 | 0.08 | 0.55 | −0.06 | 0.68 | −0.08 | 0.55 |
| BMI | −0.20 | 0.17 | 0.26 | 0.063 | −0.13 | 0.37 | −0.25 | 0.081 |
| FEV_1_ | 0.41 | 0.003 | 0.15 | 0.31 | 0.19 | 0.18 | 0.19 | 0.18 |
| FVC | 0.21 | 0.14 | 0.07 | 0.62 | 0.14 | 0.34 | 0.16 | 0.27 |
| FEV_1_/FVC | 0.50 | <0.001 | 0.09 | 0.54 | 0.13 | 0.36 | 0.12 | 0.39 |
| mMRC | −0.39 | 0.004 | −0.30 | 0.030 | −0.25 | 0.077 | −0.24 | 0.088 |
| ACT score | 0.17 | 0.22 | 0.21 | 0.15 | 0.16 | 0.28 | 0.10 | 0.47 |
| 6MWT distance | 0.38 | 0.007 | 0.16 | 0.26 | 0.22 | 0.12 | 0.21 | 0.14 |
| HADS score, depression | −0.30 | 0.035 | −0.09 | 0.53 | −0.15 | 0.31 | −0.21 | 0.15 |
| HADS score, anxiety | −0.26 | 0.070 | −0.27 | 0.058 | −0.27 | 0.057 | -0.24 | 0.094 |
| AQLQ score | 0.18 | 0.21 | 0.19 | 0.17 | 0.08 | 0.59 | 0.06 | 0.69 |

*Pearson’s correlation coefficients. 6MWT, 6-minute walk test; ACT, asthma control test; AQLQ, asthma quality of life questionnaire; BMI, body mass index; FEV_1_, forced expiratory volume in 1 s; FVC, forced vital capacity; HADS, hospital anxiety and depression scale; METs, metabolic equivalents; mMRC, modified Medical Research Council questionnaire.

Table S2. Multivariable linear regression analysis of factors affecting the total energy expenditure (kcal/day) in patients with asthma

| Factor | Estimate | SE | *P* value | Partial *R*^2^ (%) |
| --- | --- | --- | --- | --- |
| BMI | 48.0 | 15.9 | 0.004 | 7 |
| mMRC | −240.7 | 74.6 | 0.002 | 17 |

BMI, body mass index; METs, metabolic equivalents; mMRC, modified Medical Research Council questionnaire; SE, standard error.

Table S3. Multivariable linear regression analysis of factors affecting energy expenditure ≥3METs (kcal/day) in patients with asthma

| Factor | Estimate | SE | *P* value | Partial *R*^2^ (%) |
| --- | --- | --- | --- | --- |
| mMRC | −99.7 | 64.1 | 0.13 | 8 |
| HADS score, anxiety | −27.8 | 18.1 | 0.13 | 4 |

HADS, hospital anxiety and depression scale; METs, metabolic equivalents; mMRC, modified Medical Research Council questionnaire; SE, standard error.

Table S4. Multivariable linear regression analysis of factors affecting the duration of physical activity ≥3METs (min/day) in patients with asthma

| Factor | Estimate | SE | *P* value | Partial *R*^2^ (%) |
| --- | --- | --- | --- | --- |
| mMRC | −21.9 | 10.4 | 0.040 | 9 |

METs, metabolic equivalents; mMRC, modified Medical Research Council questionnaire; SE, standard error.
